# Shikonin Inhibits Inflammatory Response in Rheumatoid Arthritis Synovial Fibroblasts via lncRNA-NR024118

**DOI:** 10.1155/2015/631737

**Published:** 2015-11-10

**Authors:** Ke-ya Yang, Dong-liang Chen

**Affiliations:** ^1^Cadres' Ward, Huaihe Hospital of Henan University, Kaifeng 475000, China; ^2^Traditional Medicine Ward, Huaihe Hospital of Henan University, Kaifeng 475000, China

## Abstract

*Background*. Shikonin is a major chemical component of zicao that possesses anti-inflammatory properties and the ability to mediate cellular and humoral immunity, especially in rheumatoid arthritis (RA). We investigated the impact of shikonin on inflammatory response in RA synovial fibroblasts using the CAIA model. *Methods*. Severe polyarticular arthritis was induced in Balb/c female mice. Expressions of lncRNA-NR024118, SOCS3, proinflammatory cytokines, and MMPs were evaluated using RT-RCR. Histone acetylation and SOCS3 protein expression were assessed by ChIP assay and western blot, respectively. *Results*. Mice treated with shikonin showed an abrogation of soft tissue and bone lesions. Shikonin remarkably enhanced the expression of NR024118 and SOCS3 and suppressed the secretion and expression of IL-6, IL-8, and MMPs. Proliferation of cultured RA synovial fibroblasts in the presence of IL-1*β* was also significantly inhibited by shikonin. Moreover, shikonin dose-dependently increased acetylation of histone H3 at the promoter of NR024118. Finally, NR024118 overexpression and interference significantly changed SOCS3 expression and NR024118 interference could reverse regulation of shikonin on SOCS3, proinflammatory cytokines, and MMPs expression level in MH7A cells. *Conclusion*. Our results reveal that, in the CAIA mouse model of RA, shikonin has disease modifying activity that is attributable to the inhibition of inflammatory response via lncRNA-NR024118.

## 1. Introduction

Rheumatoid arthritis (RA) is a chronic autoimmune disease of the synovium that can lead to severe joint damage and afflicts 0.5–1.0% of population in the industrialized world [1]. RA is characterized by cellular infiltration, pannus formation, cartilage degradation, bone erosion, and extensive angiogenesis restricted to the synovium [2]. About 30% of patients became permanently work disabled during the first 2-3 years of the disease if insufficiently treated [3]. To date, we know that the pathology of RA is complex and is mediated by several mechanisms.

RA synovial fibroblasts play a critical role in the pathogenesis of RA. The remarkable increase of synovial fibroblasts in RA is accompanied by angiogenesis and infiltration of inflammatory mononuclear cells and ultimately results in pannus formation [4]. Except for soft tissue and bone lesions, RA synovial fibroblasts can spontaneously secrete numerous proinflammatory cytokines such as interleukin-6 (IL-6) and IL-8 and matrix metalloproteinases (MMPs) including MMP-1 and MMP-3, which result in infiltration of inflammatory cells and play a critical role in progressive destruction of articular cartilage and bone [5–7]. Meanwhile, as key intracellular inhibitors of cytokine signaling, SOCS3 has been thought to have profound effects on the regulation of immunity and inflammation by affecting all lineages involved in immune and inflammatory responses [8].

Long noncoding RNAs (lncRNAs) are defined as a kind of noncoding transcripts longer than 200 nucleotides and lack protein encoding capacity [9]. Although initially thought to be spurious transcriptional noise, lncRNAs are recently being considered to be molecules with diverse functional roles and mechanisms of biogenesis and function. lncRNAs as we know today regulate gene expression at various levels [10, 11].

Traditionally, it is believed that zicao (purple gromwell), the dried root of* Arnebia euchroma* (Royle) Johnst. or* Arnebia guttata* Bunge, has the ability to remove heat from the blood and detoxify [12]. It has been used as traditional Chinese herbal medicine in China for thousands of years to treat macular eruption, measles, sore-throat, carbuncle, and burn [12]. Hydroxynaphthoquinones, chemical components isolated from zicao, are the major anti-inflammatory active constituents [13, 14]. R-configuration of hydroxynaphthoquinones is identified as shikonin derivatives [13]. As the major active ingredient isolated from zicao with a molecular weight of 288, shikonin has long been used in traditional Asian medicine for burns, anal ulcers, hemorrhoids, infected crusts, external wounds, and psoriasis due to its numerous pharmacological properties [13–16], including anti-inflammatory and antitumor properties [17] as well as the ability to mediate cellular and humoral immunity [18–21]. However, the efficacy of shikonins in reducing inflammatory response in RA synovial fibroblasts via lncRNA-NR024118 has not been reported [22, 23]. Thus, we speculate that shikonin may confer anti-inflammatory action against RA. To explore its efficacy for RA, we investigated the effect of shikonin on lncRNA-NR024118 and SOCS3 expression, inflammatory cytokine, and MMPs expression and production in the anti-collagen monoclonal antibodies (CAIA) model.

## 2. Materials and Methods

### 2.1. Animals and Therapeutic Agents

Specific pathogen-free 5- to 7-week-old Balb/C female mice were purchased from the Center of Experimental Animals, Tianjin University, China. Purified shikonin (>98%) was purchased from Sigma-Aldrich (S7576) which was dissolved in 2% dimethyl sulfoxide (DMSO) from Sigma-Aldrich (St. Louis, MO, USA) [24] to prepare concentrations of 0, 0.1, 0.5, and 1.0 mg/kg. In particular, shikonin concentrations of 0 mg/kg were considered to be dissolved in vehicle control. All experimental procedures were performed strictly in adherence to the Guide for Laboratory Animals and Care of the Institute of Laboratory Animal Resources, National Academy of Sciences, National Research Council, and were examined and approved by the Institutional Animal Care and Use Committee at Huaihe Hospital of Henan University.

### 2.2. Induction of CAIA by Anticollagen Monoclonal Antibodies and LPS

The induction of CAIA and preparation of four monoclonal antibodies were performed as described previously [25] and arthritis in this model is mediated by immune complex mediated complement activation [26]. Briefly, two mg of Arthrogen-CIA per mouse was injected intravenously into the tail vein of mice. Subsequently, mice were given intraperitoneal injection with 25 *μ*g of LPS (*E. coli* strain 0111:B4) twenty-four hours later. And 24 hrs following LPS challenge, mice were treated with shikonin (0, 0.1, 0.5, and 1.0 mg/kg, p.o., daily) for 10 days. The extent of disease was scored in a blinded fashion by visual observation according to the reference [25].

### 2.3. Quantitative Histomorphometric Analysis

Preparation of paws and toluidine blue stain for Quantitative Histomorphometric Analysis was referred to in [20]. The significance of the arthritic score was assessed on study day 10. Osteomeasure software (Osteometer, Atlanta, GA) was used to quantify disease manifestation in the stained sections. Percentage of damaged articular surface, percentage of articular area without proteoglycan staining, thickness of articular cartilage, thickness of articular cartilage without proteoglycan staining, and osteoclast number per marrow area either were obtained directly by tracing and dotting or were calculated.

### 2.4. Isolation and Culture of Synovial Cells

Synovial fibroblasts were isolated on study day 10 by sequential digestion of the dissected synovial tissues with type I collagenase and cultured in Dulbecco Modified Eagle Medium (DMEM) (Gibco, Grand Island, NY, USA) at 37°C in a humidified atmosphere with 5% CO_2_, supplemented with 10% (v/v) fetal bovine serum.

### 2.5. Cell Proliferation Assay

For cell proliferation assay, synovial fibroblasts were isolated from 5 patients with RA, who were all female with mean age 62.0 ± 2.4. C-reactive protein in serum was 5.2 ± 1.7 (mg/dL) and erythrocyte sedimentation rate was 27.6 ± 5.4 (mm/hour). Diagnosis of RA was carried out as described previously [27]. Shikonin was dissolved in vehicle (2% DMSO) to prepare concentrations of 0, 1, 5, and 10 *μ*M as above. Synovial fibroblasts were plated in 96-well plates at a density of 2.5 × 10^3^ cells per well and allowed to synchronize by incubation in serum-free DMEM medium for 48 h. Then, the cell culture medium was replaced by normal DMEM medium containing shikonin at varying concentrations. Cells were counted at 0, 24, 48, and 72 h and cell number was evaluated by crystal violet staining [28].

### 2.6. MH7A Synovial Cell Cultures

MH7A synovial cells were purchased from Jennio Biotech Co., Ltd. (Guangzhou, China). The cell line was established by transfection with the SV40 T antigen [29]. MH7A cells were grown in RPMI-1640 medium (Gibco BRL) supplemented with 10% heat-inactivated FBS (Gibco BRL), penicillin (final concentration, 100 U/mL), and streptomycin (final concentration, 0.1 mg/mL) at 37°C in 5% CO_2_ humidified atmosphere.

### 2.7. Microarray Analysis

The expression profile of long noncoding RNAs and mRNAs in synovial fibroblasts was analyzed by using the rat lncRNA 4 × 44 K Arrays from Arraystar (Arraystar, Rockville, USA). The microarray hybridization was performed as described previously [30]. In brief, total RNA was isolated with TRIzol reagent (Invitrogen, Carlsbad, CA, USA) according to the supplier's instruction. Then, 1 *μ*g of total RNAs was amplified and transcribed into fluorescent cRNA using Agilent's Quick Amp Labeling protocol (version 5.7, Agilent Technologies, USA). Subsequently, the labeled cRNAs were hybridized onto the rat lncRNA 4 × 44 K Array, which includes 9300 lncRNAs and 15,200 coding transcripts. After washing the slides, the arrays were scanned using an Agilent G2505B Scanner. Acquired array images were extracted using Agilent Feature Extraction Software (version 10.7.3.1). The GeneSpring GX v11.5.1 software package (Agilent Technologies) was used to perform quantile normalization and subsequent data processing.

### 2.8. Real-Time PCR (RT-PCR)

Hind limbs, including the ankle joint, were removed and snap-frozen in liquid nitrogen. Quantitative real-time PCR (qPCR) was performed to quantify the levels of lncRNA-NR024118 and expression of SOCS3, IL-6, IL-8, MMP-1, and MMP-3. Firstly, total RNA was isolated using TRIzol reagent (Invitrogen) and reversely transcribed to cDNA using a RevertAid cDNA Synthesis Kit (Fermentas International Inc., Vilnius, Lithuania) according to the manufacturer's instructions. Then, real-time PCR was performed using the ABI7300 Sequence Detection systems (Applied Biosystems, CA, USA). cDNAs were amplified from 3 to 5 *μ*L of the cDNA reaction mixture using specific gene primers. The primer sequences and reaction conditions of NR024118, SOCS3, IL-6, IL-8, MMP-1, MMP-3, and *β*-actin were shown in attached Table 1. Gene expression in each sample was normalized to actin expression.

### 2.9. Western Blot Analysis

Cultured cells were treated as above and lysed in 20 *μ*L of cell lysis buffer containing 1 mm phenylmethanesulfonyl fluoride (PMSF). Samples from these cell lysates were denatured and subjected to 17% SDS-PAGE. Proteins were transferred to PVDF membranes by 2 h electroblotting. Membranes were blocked in 5% nonfat dry milk for 1 h at RT and then incubated overnight at 4°C with primary antibodies. Following each incubation, the membrane was washed extensively with TBS containing 0.05% Tween-20 (TBST buffer) three times, probed overnight with anti-SOCS3 (Abcam, CA, USA) and anti-*β*-actin (Cell Signaling Technology, MA, USA), and then incubated with horseradish peroxidase-conjugated secondary antibodies (Boster, Wuhan, China) for 1 h. Finally, the blots were detected by the enhanced chemiluminescence (ECL) kit (Amersham Biosciences, Piscataway, NJ) [28].

### 2.10. ChIP Histone Acetylation

Histone acetylation was determined by real-time PCR analysis of chromatin immunoprecipitates (ChIP) from RA synovial fibroblasts. Fresh cultures of RA synovial fibroblasts were collected, treated with shikonin (0, 0.1, 0.5, and 1.0 M), and fixed with formaldehyde. As a substrate for immunoprecipitation with antibodies to acetyl-H3 (Ac-H3) or acetyl-H4 (Ac-H4), chromatin was then isolated and sheared by sonication. Finally, the total DNA was isolated from reversibly cross-linked chromatin and subsequently subjected to real-time PCR.

### 2.11. Plasmid Construction and Cell Transfection

The NR024118 sequence was synthesized and subcloned into pCDNA3.1 vector (Invitrogen, Shanghai, China). The pCDNA-NR024118 or empty vector was transfected into MH7A cells cultured in six-well plates according to the manufacturer's protocol. Transfection of the empty vector pcDNA3.1 was used as the control. Meanwhile, the siRNAs used for NR024118 knockdown were also, respectively, transfected into MH7A cells. Cells fusion and transfection were carried out using lipofectamine 2000 (Invitrogen, USA) as recommended by the manufacturer's instructions. At 48 h after transfection, cells were harvested and lysed for RT-PCR or western blot analysis.

### 2.12. Statistical Analysis

The experimental data were determined by Dunnett's *t*-test for comparisons of arthritic scores and real-time PCR quantitation. At study day 10, histomorphometric analysis was performed by combining right and left hind paws from individual animals and parameters were analyzed by Student's *t*-test through GraphPad Prism Version 5.0 software (GraphPad Software Inc., San Diego, CA, USA). Cell viability as well as cytokines/MMPs expression and secretion was also analyzed by Student's *t*-test. The statistical analyses were conducted using SAS statistical analysis software (SAS Institute, Cary, NC) and data were presented as mean ± SD. *P* values were determined by *t*-test and the statistically significant difference was considered at *P* < 0.05 or *P* < 0.01.

## 3. Results

### 3.1. Shikonin Treatment Dose-Dependently Decreases Arthritic Score and Inhibits Parameters of Disease Progression in a Mouse Model of Rheumatoid Arthritis

**Figure 1:**
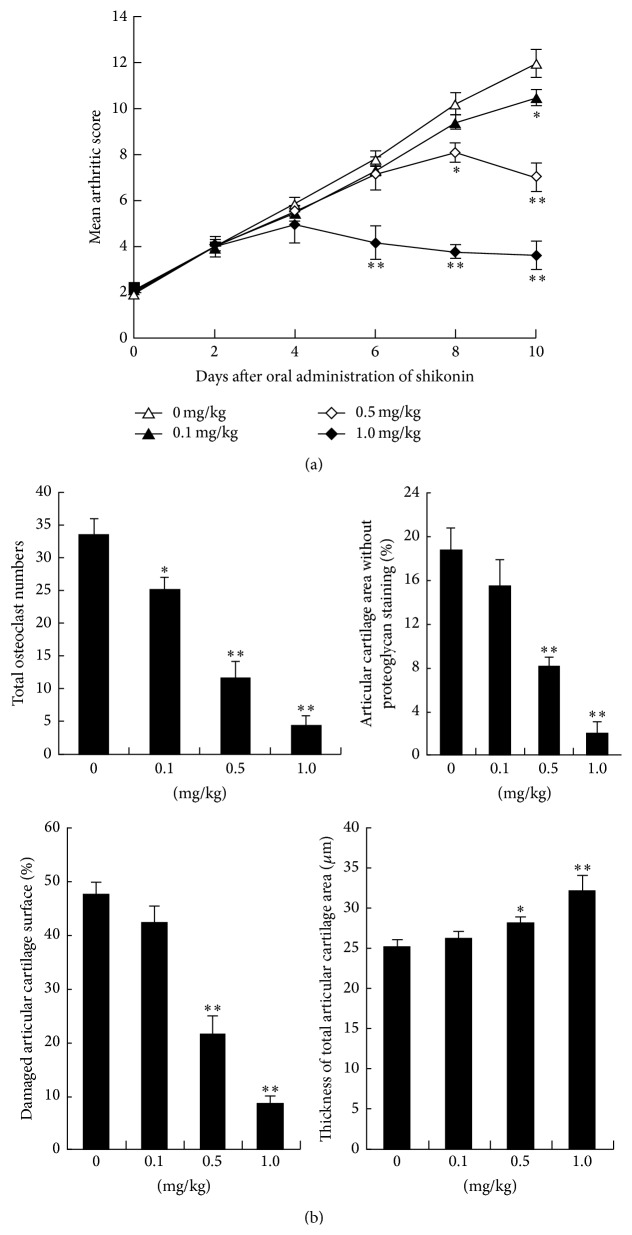
Daily gavage with shikonin decreases arthritic score and inhibits parameters of disease progression in an antibody-induced model of rheumatoid arthritis. Female Balb/c mice were treated as described in Materials and Methods to construct CAIA model and then orally administered shikonin treatment at 0, 0.1, 0.5, or 1.0 mg/kg for 10 consecutive days. Arthritic score of mice was assessed on study days 2, 4, 6, 8, and 10. Maximum arthritic score is 16 for each mouse (a). Blinded samples were used to count osteoclast number using acid phosphatase/toluidine blue stained slides and the articular area and surface were performed using Safranin O stained slides. Ostemeasure software, Nikon Eclipse E400 light/epiflourescent microscope, and video system were applied in histomorphometric measurements (b). Each value was expressed as mean ± SD. ^*∗*^ *P* < 0.05, ^*∗∗*^ *P* < 0.01 versus control.

The collagen antibody-induced arthritis model in mice was employed in investigation of the antiarthritic effect of shikonin. In this model, mice orally treated with shikonin were assessed on study days 2, 4, 6, 8, and 10 to evaluate arthritic score. While CAIA model mice showed a significant increase in clinical scores and obvious soft tissue and bone lesions as compared to healthy mice (data not shown), shikonin could dose-dependently reduce clinical scores (Figure 1(a)), total osteoclast numbers, articular cartilage area without proteoglycan staining, and damaged articular cartilage surface and increase thickness of total articular cartilage area on study day 10 (Figure 1(b)).

### 3.2. Shikonin Dose-Dependently Upregulates lncRNA-NR024118 and SOCS3 Expression and Reduces Gene Expression of Inflammatory Cytokines and Matrix Metalloproteinases (MMPs)

**Figure 2:**
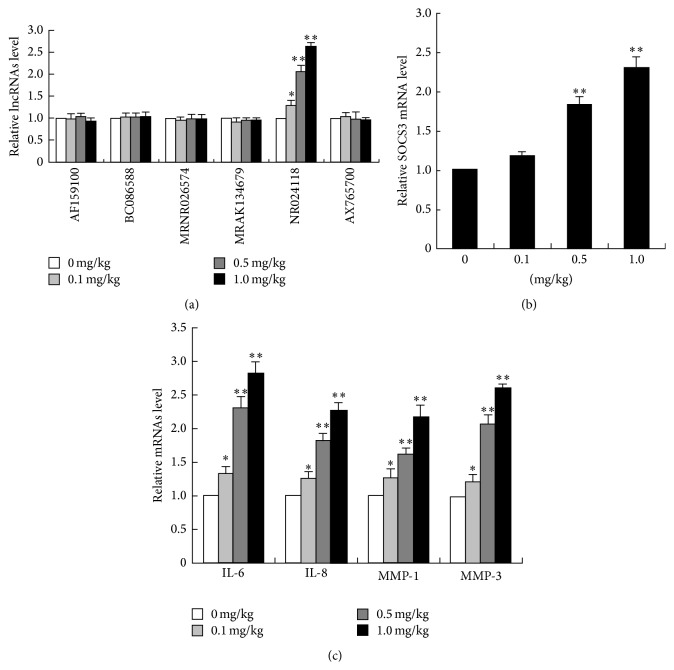
Effect of shikonin on the expression of lncRNA-NR024118, SOCS3, inflammatory cytokines, and MMPs in synovial fibroblasts. On study day 10, hind limbs from mice treated with shikonin were harvested and homogenized. Synovial fibroblasts were isolated from these samples, total RNA was extracted, and then the expression of lncRNAs in synovial fibroblasts from mice treated with shikonin was determined as demonstrated by the microarray analysis (a). The mRNA levels of SOCS3 (b), IL-6, IL-8, MMP-1, and MMP-3 (c) were determined with RT-RCR. The data are presented as the mean ± SD of five determinations. ^*∗*^ *P* < 0.05, ^*∗∗*^ *P* < 0.01 versus control.

To investigate the mechanism of the anti-inflammatory effects of shikonin in this murine model of RA, the changes in the expression of various genes were examined by a microarray analysis after shikonin treatments of the synovial fibroblasts. Present results revealed that there were a total of six genes related to the development and procession of RA in which a quantitative fluctuation of expression was observed (Figure 2(a)). Among these immunological-related genes, the most strongly upregulated gene was lncRNA-NR024118. Therefore, we chose NR024118 for further studies. Moreover, shikonin treatment dose-dependently increased gene expression of NR024118, SOCS3 (Figure 2(b)), IL-6, IL-8, MMP-1, and MMP-3 (Figure 2(c)) in the joints of CAIA mice when compared with the control. These data revealed that a local increase with shikonin administration results in an increase in lncRNA-NR024118 and SOCS expression which correlated with suppression of arthritis development.

### 3.3. Shikonin Dose- and Time-Dependently Inhibits the Proliferation of Synovial Fibroblasts and Reverses the Production of Proinflammatory Cytokines and MMPs and the Expression of SOCS3 and lncRNA-NR024118 Reduced by IL-1*β*

**Figure 3:**
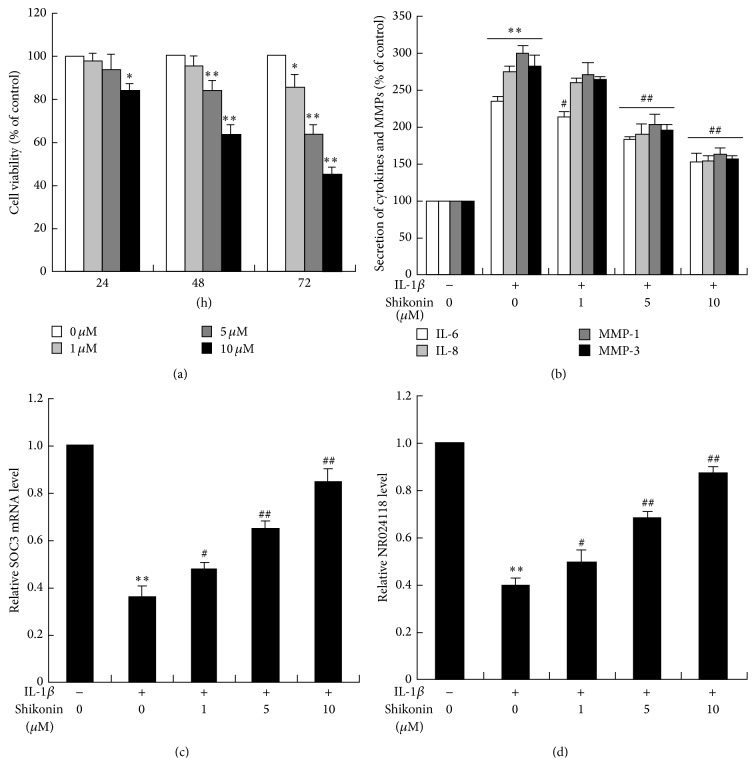
Effect of shikonin on the proliferation of RA synovial fibroblasts and production of proinflammatory cytokines and MMPs. Cells were synchronized and incubated with shikonin at various concentrations for 24, 48, and 72 h, respectively. Data were expressed as the percentage relative to the untreated control samples, and each value was expressed as mean ± SD for three independent experiments by GraphPad Prism Software Version 5.0 (a). Synovial fibroblasts in the presence of 10 ng/mL IL-1*β* induction were treated with shikonin at various concentrations (0, 1, 5, and 10 *μ*M) for 24 h in serum-free DMEM medium. After treatments, the cell-free medium and RA synovial fibroblasts were collected to evaluate the secretion of IL-6, IL-8, MMP-1, and MMP-3 by their corresponding ELISA kits, respectively (b), and the expression of SOCS3 (c) and NR024118 (d) was determined by RT-RCR. ^*∗*^ *P* < 0.05, ^*∗∗*^ *P* < 0.01 versus control. ^#^ *P* < 0.05, ^##^ *P* < 0.01 versus IL-1*β*.

The effect of shikonin on the proliferation of synovial fibroblasts from RA patients was evaluated by crystal violet staining. As shown in Figure 3(a), shikonin repressed the proliferation of synovial fibroblasts in a dose- and time-dependent manner. Furthermore, the application of shikonin resulted in a dose-dependent reduction on the secretion of IL-6, IL-8, and MMP-3 (Figure 3(b)) and increase on the expression of SOCS3 and NR024118 (Figures 3(c) and 3(d)) in synovial fibroblasts induced by IL-1*β*.

### 3.4. Shikonin Increases Acetylation of Histone H3 at the Promoter of lncRNA-NR024118 in RA Synovial Fibroblasts in a Dose-Dependent Manner

**Figure 4:**
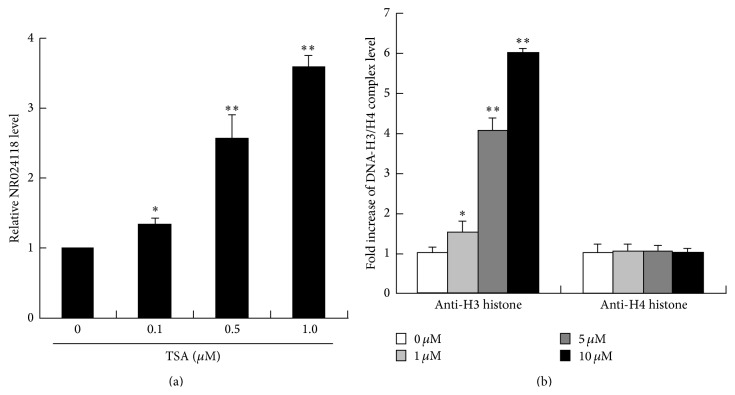
Effect of TSA on the expression of NR024118 and the application of Shikonin on histone acetylation at NR024118 promotor. Synovial fibroblasts from RA patients were treated with TSA (0, 0.1, 0.5, and 1.0 *μ*M) for 24 h. Gene expression of NR024118 was determined by RT-RCR and all RNA values were normalized to *β*-actin mRNA expression. This experiment was repeated three times (a). Histone acetylation was determined by real-time PCR analysis of chromatin immunoprecipitates from RA synovial fibroblasts. Real-time PCR values were determined by reference to a standard curve generated by real-time PCR amplification of genomic DNA using NR024118 promoter primers. Each value was normalized to the total amount of NR024118 promoter DNA added to the immunoprecipitation reaction. Fold increases compared to untreated cells are shown; representative data from at least three separate chromatin preparations are shown (b). ^*∗*^ *P* < 0.05, ^*∗∗*^ *P* < 0.01 versus control.

Our data further demonstrated that Trichostatin A (TSA) significantly increases NR024118 expression level in RA synovial fibroblasts and affected expression of NR024118 in a dose-dependent manner (Figure 4(a)). What is more, shikonin increased acetylation of H3 at the NR024118 promoter in RA synovial fibroblasts in a dose-dependent manner, whereas little to no fluctuation of H4 acetylation was detected at various shikonin concentrations (Figure 4(b)).

### 3.5. NR024118 Overexpression and Interference Significantly Changed Expression of SOCS3 in MH7A Cells

**Figure 5:**
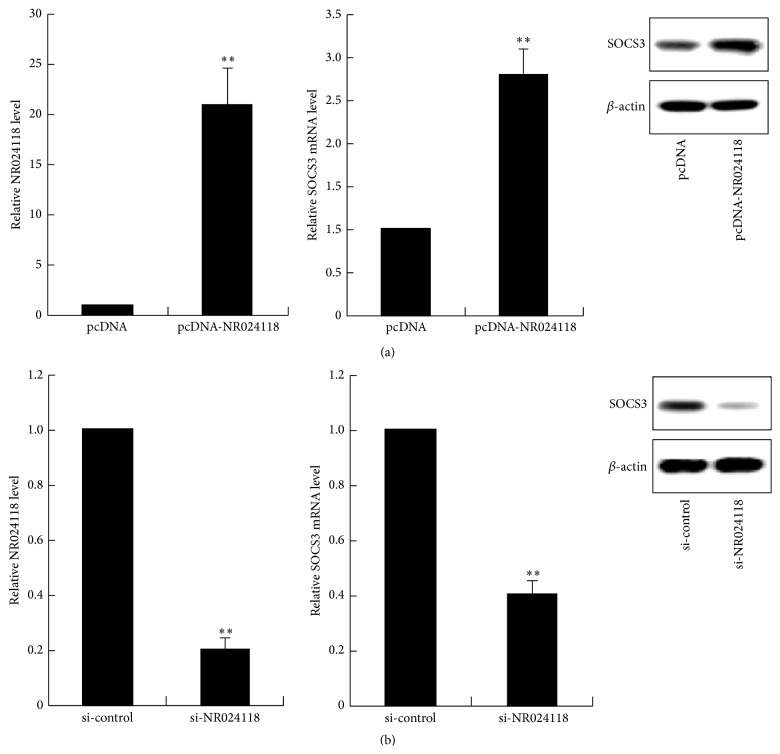
The level of NR024118 and SOCS3 expression in MH7A cells. Real-time PCR analysis of NR024118 and SOCS3 expression levels following the treatment of MH7A cells with pCDNA-NR024118 and empty vector. SOCS3 protein expression in MH7A cells treated with pCDNA-NR024118 was assessed by western blot. *β*-actin protein expression was used as an internal control (a). RT-PCR analyses of NR024118 and SOCS3 expression level following treatment MH7A cells with si-NR024118 or si-control. SOCS3 protein expression in MH7A cells treated with si-NR024118 was also assessed by western blot (b). ^*∗∗*^ *P* < 0.01 versus control.

RT-RCR analysis of NR024118 levels revealed that NR024118 expression was significantly increased in pCDNA-NR024118 transfected cells and significantly decreased in si-NR024118 transfected cells (Figure 5). RT-RCR and western blot assays were performed to detect the expression of SOCS3 mRNA and SOCS3 protein in MH7A cells. As shown in Figure 5(a), the expression level of SOCS3 mRNA and protein increased significantly in pCDNA-NR024118 group compared with the control group. However, the expressions of SOCS3 were reduced markedly when the expression of NR024118 was knocked down (Figure 5(b)).

### 3.6. NR024118 Interference Can Recover the Effect of Shikonin Upregulation on mRNA Level of SOCS3 and Downregulation on SOCS3 Protein, Proinflammatory Cytokines, and MMPs Expression Level in MH7A Cells

**Figure 6:**
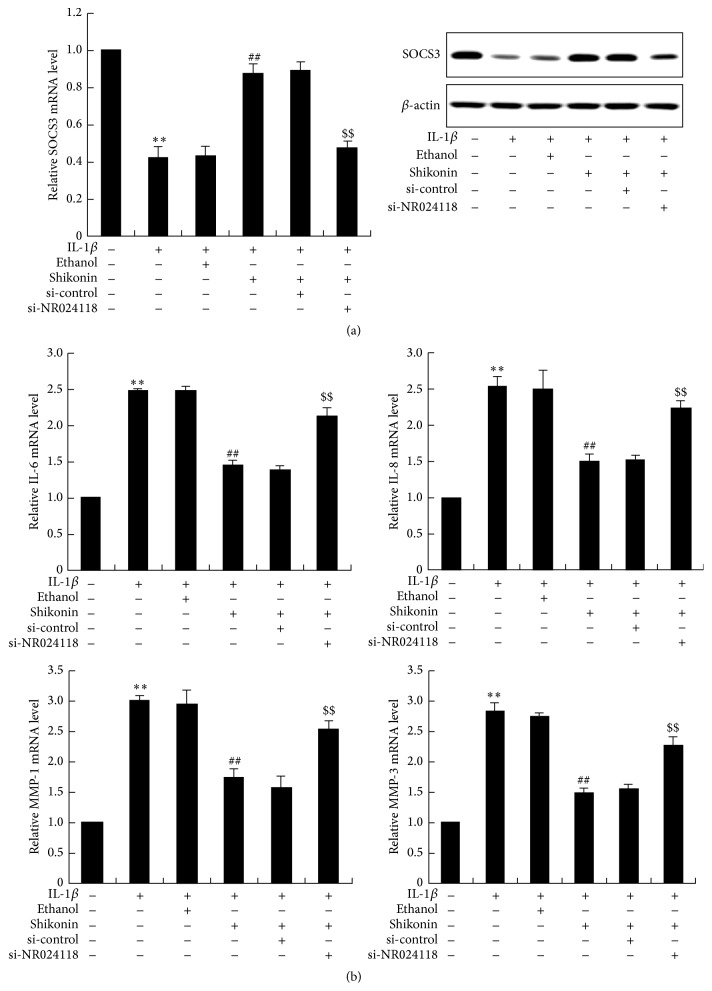
Detection of SOCS3, IL-6, IL-8, MMP-1, and MMP-3 expression in NR024118 interfered MH7A cells. After being treated with 4 *μ*M shikonin and normal incubation for 24 hours, enhanced SOCS3 expression levels were observed in IL-1*β* induced MH7A cells while this change could be recovered by NR024118 interference (a). When MH7A cells were treated with 4 *μ*M shikonin, diminished IL-6, IL-8, MMP-1, and MMP-3 were detected, and this change could also be reversed by NR024118 interference (b). ^*∗∗*^ *P* < 0.01 versus control. ^##^ *P* < 0.01 versus ethanol. ^$$^ *P* < 0.01 versus si-control.

To explore effect of shikonin treatment and NR024118 interference on SOCS3, proinflammatory cytokines, and MMPs expression, MH7A cells transfected with si-NR024118 or si-control were treated with 4 *μ*M shikonin. We confirmed that 4 *μ*M shikonin treatment had no significant effect on cell viability of MH7A cells. Moreover, the decrease of SOCS3 mRNA and protein expression levels induced by IL-1*β* were attenuated by the presence of shikonin (4 *μ*M) in MH7A cells. Interestingly, this change could be further reversed by NR024118 interference (Figure 6(a)). On the other hand, shikonin treatment could reverse IL-1*β* induced abundance of IL-6, IL-8, MMP-1, and MMP-3 expression in MH7A cells. Similarly, the reduction of them was also rescued by following NR024118 interference (Figure 6(b)).

## 4. Discussion

In this paper, our data demonstrated that the oral administration of shikonin in a mouse collagen antibody-induced model of RA led to a dose-dependent inhibition of arthritic disease. Shikonin could be a disease modifying agent for RA and possesses positive effects in RA such as suppression of proliferation synovial fibroblasts, increase of lncRNAs and SOCS3 expression, and inhibition of cytokines expression and production.

As a pathogenesis of autoimmune disease, RA has been recently shown to be associated with various lncRNAs [31–35]. We found that NR024118 was strongly upregulated by shikonin in RA synovial fibroblasts (Figures 2(a) and 3(d)). Exciting research from around the world in the very recent years implicates that lncRNA-NR024118 was widely involved in pathophysiology of diseases, including human cancer, cardiovascular diseases, neural diseases, and rheumatoid arthritis (RA) [30, 36]. NR024118 may be regulated by shikonin, as indicated in this study, and appears to play a role in pathogenesis of RA. In fact, present data showed that shikonin dose-dependently increased acetylation of histone H3 at the promoter of lncRNA-NR024118 (Figure 4(b)). These results demonstrated that shikonin may inhibit the synovial fibroblasts proliferation and attenuate soft tissue and bone lesions via the expression of NR024118.

As a major regulator of CD4+ T lymphocyte activation, SOCS proteins have also been shown to downregulate the responses of immune cells to cytokines [37]. One recent study showed that chronically and granulomatously inflamed human tissues revealed higher levels of SOCS3 expression in inflamed than in corresponding normal tissues [38]. Moreover, SOCS3 was upregulated in monocytes in the peripheral blood and synovial fluid of RA patients [39]. In this study, SOCS3 mRNA expression was significantly enhanced by shikonin (Figures 2(b) and 3(c)). NR024188 overexpression and interference significantly changed expression of SOCS3 mRNA and proteins, and NR024118 interference could recover the effect of shikonin upregulation on SOCS3 mRNA and protein expression level in MH7A cells (Figures 5 and 6(a)).

Inflammatory changes of synovial fibroblasts have been thought to play a vital role in the progression of RA. Meanwhile, most of SOCS proteins were thought to be induced by cytokines and therefore act in a classical negative-feedback loop to inhibit cytokine signal transduction [40]. Though it has been reported that IL-6 and IL-8 were overexpressed in infected synovial fibroblasts, the effect of IL-6 on the proliferation of synovial fibroblasts was in dispute [41, 42]. In addition to abnormal apoptotic regulation in synovial fibroblasts, several studies have shown that MMPs are involved in the irreversible destruction of cartilage and bone process in RA, which are pivotal in the recruitment of leukocytes and macrophages into joints [43, 44]. In particular, two recent studies on collagen-induced arthritis (CIA) that examined the effect of shikonin on inflammatory cytokines demonstrated that shikonin significantly inhibited the production of MMP-1 and Th1 cytokines expression and elevated tissue inhibitors of metalloproteinase- (TIMP-) 1 and Th2 cytokines expression in mice with CIA [22, 45]. In our study, we observed that shikonin could dose-dependently reduce the expression of IL-6, IL-8, MMP-1, and MMP-3, significantly decrease the secretion of IL-6 and IL-8, and exert immunological effects, including decreasing MMPs production, if excessive proinflammatory cytokines are present under pathological conditions (Figures 2(c) and 3(b)). Besides, we also found that NR024118 interference could reverse the effect of shikonin upregulation on IL-6, IL-8, and MMPs expression level in MH7A cells (Figure 6(b)). Recent studies have revealed that IL-6 and IL-8 are key mediators that significantly promote the synthesis and production of MMPs and shikonin inhibition of IL-6 and IL-8 may be partly responsible for the decrease of MMPs secretion [28]. Hence, shikonin may suppress hyperplasia of the synovial membrane and the subsequent invasion and destruction of the adjoining cartilage by the activation of locally produced MMPs.

Many previous studies have been carried out to investigate the progressive destruction of the structural components of the joints in the progression of RA. In this murine CAIA model, the effect of shikonin treatment on cartilage and bone destruction was also studied and shikonin treatment demonstrated a dose-dependent decrease in mean arthritic scores and inhibition of ablated osteoclast number as determined by TRAP staining, when compared to control-treated mice (Figure 1). By measuring proteoglycan loss in further investigation, results revealed the sparing of articular cartilage damage and maintenance of total cartilage thickness. A similar study indicated that shikonin was involved in cartilage protection since it significantly reduced the incidence and severity of CIA and markedly abrogated joint swelling and cartilage destruction [22]. Moreover, another interesting research aiming to investigate the effect of shikonin on early stage and established murine CIA showed that shikonin (5 mg/kg) treatment along had no effect on macroscopic score and incidence of arthritis on early stage of CIA [45]. However, when treated with shikonin on established CIA for 10 days, mice presented pronounced amelioration of macroscopic score and cartilage destruction. In our study, we have also found that treatment with shikonin is associated with an inhibition in the expression of IL-6, IL-8, MMP-1, and MMP-3 in RA synovial fibroblasts. Therefore, in this mouse collagen antibody-induced model of RA, shikonin strikingly attenuated inflammatory response and markedly inhibited tissue destruction observed by elevating the expression of SOCS3 and suppressing production of inflammatory cytokines and matrix metalloproteinases via lncRNA-NR024188.

## 5. Conclusion

Based on our comprehensive and systematic exploration in cultured RA synovial fibroblasts, shikonin may confer anti-inflammatory action against RA in a mouse collagen antibody-induced model through abrogating soft tissue and bone lesions, suppressing synovial fibroblasts proliferation, and inhibiting the expression and secretion of proinflammatory cytokines and MMP. We further investigate whether shikonin regulates the inflammatory response via lncRNA-NR024118. To sum up, due to its anti-inflammatory effect in pathophysiological processes of RA, shikonin may have promising potential for the development of an attractive and suitable therapeutic agent for RA.

## Supplementary Material

To investigate the effects of shikonin treatment and lncRNA-NR024118 interference on SOCS3, proinflammatory cytokines and MMPs expression, MH7A cells transfected with si-NR024118 or si-control were treated with 4 μM shikonin. We confirmed that 4 μM shikonin treatment had no significant effect on cell viability of MH7A cells. Subsequently, the expression levels of SOCS3, proinflammatory cytokines and MMPs were evaluated.

## Figures and Tables

**Table 1:** Primer sequences and reaction conditions of RT-PCR.

Genes	Primers	Sequences (5′-3′)	Annealing temperature (°C)	Cycle number
NR024118	Forward	5′GCTGCCCACCTCACTCAC3′	63°C	40
	Reverse	5′CTTTATTGCTCCATTTCCCTC3′		

SOCS3	Forward	5′TTCTTCACGCTCAGCGTCAAG3′	55°C	30
	Reverse	5′ATGTAATAGGCTCTTCTGGGG3′		

IL-6	Forward	5′GGCTGCTTCTGGTGATGG3′	55°C	30
	Reverse	5′AGAGATTTTGCCGAGGATGTA3′		

IL-8	Forward	5′GCCAAGGAGTGCTAAAGAACTTAGA3′	58°C	30
	Reverse	5′ATTTCTGTGTTGGCGCAGTGT3′		

MMP-1	Forward	5′AGGGTCAAGCAGACATCA3′	56°C	30
	Reverse	5′CAGAAGGGCAAGCATTAG3′		

MMP-3	Forward	5′CCTGCTTTGTCCTTTGATGC3′	55°C	30
	Reverse	5′TGAGTCAATCCCTGGAAAGTC3′		

*β*-actin	Forward	5′TGACGGGGTCACCCACACTGTGCCCATCT3′	60°C	30
	Reverse	5′CTAGAAGCATTTGCGGTGGACGATG3′		
