# Supplementary figures and images for: Shikonin Inhibits Inflammatory Response in Rheumatoid Arthritis Synovial Fibroblasts via lncRNA-NR024118
Source: Evid Based Complement Alternat Med. 2015 Nov 10;2015:631737. doi: 10.1155/2015/631737 (PMC4657066; doi:10.1155/2015/631737)

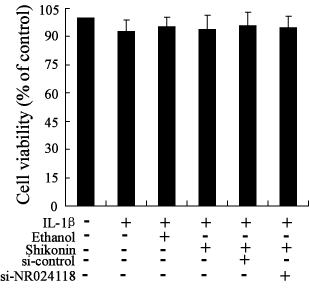

Supplement: Supplementary file 1 — To investigate the effects of shikonin treatment and lncRNA-NR024118 interference on SOCS3, proinflammatory cytokines and MMPs expression, MH7A cells transfected with si-NR024118 or si-control were treated with 4 μM shikonin. We confirmed that 4 μM shikonin treatment had no significant effect on cell viability of MH7A cells. Subsequently, the expression levels of SOCS3, proinflammatory cytokines and MMPs were evaluated. [file 631737.f1.zip › 631737.f1/631737.f1.jpg]
